# Investigating white matter development in infancy and early childhood using myelin water faction and relaxation time mapping

**DOI:** 10.1016/j.neuroimage.2012.07.037

**Published:** 2012-11-15

**Authors:** Sean C.L. Deoni, Douglas C. Dean, Jonathan O'Muircheartaigh, Holly Dirks, Beth A. Jerskey

**Affiliations:** a Advanced Baby Imaging Lab, School of Engineering, Brown University, Providence, RI, USA; b Department of Human Behaviour and Psychiatry, Warren Alpert Medical School, Brown University, Providence, RI, USA; c Department of Neuroimaging Sciences, King's College London, Institute of Psychiatry, London, UK

**Keywords:** Brain development, White matter development, Infant imaging, Myelin, Myelin water fraction, Quantitative T_1_ and T_2_

## Abstract

The elaboration of the myelinated white matter is essential for normal neurodevelopment, establishing and mediating rapid communication pathways throughout the brain. These pathways facilitate the synchronized communication required for higher order behavioral and cognitive functioning. Altered neural messaging (or ‘disconnectivity’) arising from abnormal white matter and myelin development may underlie a number of neurodevelopmental psychiatric disorders. Despite the vital role myelin plays, few imaging studies have specifically examined its maturation throughout early infancy and childhood. Thus, direct investigations of the relationship(s) between evolving behavioral and cognitive functions and the myelination of the supporting neural systems have been sparse. Further, without knowledge of the ‘normative’ developmental time-course, identification of early abnormalities associated with developmental disorders remains challenging. In this work, we examined the use of longitudinal (T_1_) and transverse (T_2_) relaxation time mapping, and myelin water fraction (MWF) imaging to investigate white matter and myelin development in 153 healthy male and female children, 3 months through 60 months in age. Optimized age-specific acquisition protocols were developed using the DESPOT and mcDESPOT imaging techniques; and mean T_1_, T_2_ and MWF trajectories were determined for frontal, temporal, occipital, parietal and cerebellar white matter, and genu, body and splenium of the corpus callosum. MWF results provided a spatio-temporal pattern in-line with prior histological studies of myelination. Comparison of T_1_, T_2_ and MWF measurements demonstrates dissimilar sensitivity to tissue changes associated with neurodevelopment, with each providing differential but complementary information.

## Highlights

► Investigate brain T_1_, T_2_ and myelin water fraction development across childhood ► Establish protocols for non-sedated infant and toddler imaging ► Regional T_1_, T_2_ and MWF trajectories spanning the first 5 years of life ► Comparison between, T_1_, T_2_ and MWF estimates across the age range

## Introduction

Increasingly, many psychiatric disorders are being considered within the context of a neurodevelopmental disorder. These include disorders such as autism, attention deficit disorder and schizophrenia (Courchesne et al., 2011; Grossman et al., 2003; Wolff et al., 2012), which potentially arise from atypical brain development over the first few years of life. Aberrant white matter development and myelin maturation may lead to reduced brain connectivity (termed ‘disconnectivity’) or dis-synchronous brain messaging, which is observed in a variety of neuropsychiatric disorders (Just et al., 2012; Kana et al., 2011; Meda et al., 2012; Mills et al., 2012). While several maturation processes are associated with early neurodevelopment, including axonal migration, dendritic sprouting, synapse generation, and axonal pruning, the elaboration of the myelinated white matter through the wrapping of the fatty myelin sheath around neuronal axons (myelination), is directly involved in establishing and maintaining efficient brain communication. Thus, this process is of particular relevance to neurodevelopmental disorders. The close spatio-temporal association believed to exist between the evolution of cognitive and behavioral functioning, and the myelination of sub-serving or supporting brain networks (Johnson and Munakata, 2005) suggests that abnormal myelination may be associated with, or result in, abnormal functioning and behavior. This is further supported by the loss of functioning noted in demyelinating disorders, such as multiple sclerosis.

Evidence for a close relationship between cognitive maturation and the myelination of underlying neural systems comes principally from indirect association studies combining post-mortem studies of myelination and neurobehavioral studies on healthy infants (Melina, 2004; Rabinowics, 1986). Unfortunately, post-mortem data may not reflect typical or healthy neurodevelopment, and these studies can only draw indirect conclusions. Magnetic resonance imaging (MRI) affords a more direct means of examining these relationships in the same infants by, for example, associating changes in MRI metrics (e.g. white or gray matter ‘density’, cortical thickness, diffusion anisotropy, or relaxation time) with measures of functional or behavioral performance (Bava et al., 2011; Giedd and Rapoport, 2010; Giedd et al., 1996; Giorgio et al., 2008; Paus et al., 1999; Raznahan et al., 2011; Shaw et al., 2006; Sowell et al., 2002). However, while informative, these MRI metrics are non-specific, and reflect broad microstructural changes that make informative interpretation challenging. Further, few studies have investigated associations across the entirety of early childhood development, from infancy through to 5 years of age, instead focusing on either early development (under 1 year of age; Dubois et al., 2006; Symser et al., 2011), or older childhood (beyond 5 years of age; Giedd et al., 1996; Luna et al., 2011).

The gradual onset of ‘adult-like’ gray and white matter tissue contrast, driven by changes in the longitudinal and transverse relaxation times (T_1_ and T_2_), has been used to stage brain development (Barkovich et al., 1988; Paus et al., 2001), and proposed as a means of grading myelination (Bird et al., 1989; Pujol et al., 2004; Staudt et al., 1994). However, T_1_ and T_2_ are sensitive not only to the arrival of myelin precursory proteins and the establishment of the myelin sheath, but also to changes in bulk water content and compartmentalization (through changes in axon fiber size, density or coherence), iron content, membrane permeability, and cholesterol content (MacKay et al., 2009). By association, structural measures derived from T_1_ or T_2_ tissue contrast, including white matter density and cortical thickness, suffer similar non-specificity. Thus, caution should be used in interpreting increased white matter density as reflecting increased myelination as has been used previously (Pujol et al., 2004).

The sensitivity of diffusion-tensor (DT)-MRI to gross fiber architecture has made DT-MRI a popular choice for investigating white matter maturation (Bashat et al., 2007; Bosnell et al., 2008; Moseley, 2002; Nagy et al., 2004), with metrics including fraction anisotropy (FA), mean diffusivity (MD), axial (AD) and radial (RD) diffusivity among others, providing complementary indices of neural maturation (Bockhorst et al., 2008; Cheung et al., 2008; Snook et al., 2005). AD and RD describe the diffusivity along the principal axis, and the mean perpendicular diffusivity, respectively. Changes in these metrics are often attributed to myelin content or, more broadly, white matter integrity, however, significant FA and RD are noted in non-myelinated ex vivo nerve (Beaulieu, 2002), as well as in the frontal lobe regions of 1 week-old infants, where myelin is not yet histologically present (Provenzale et al., 2007). Thus, these measures more likely characterize the local fiber architectural milieu (fiber coherence, density, order, and size), and care should be taken when ascribing observed changes to any specific microstructural attribute.

In addition to the low specificity of measures, few imaging studies have spanned the most rapid and dynamic stage of neurodevelopment: birth through 5 years of age (Almli et al., 2007; Hagmann et al., 2010; Huang et al., 2006; Leppert et al., 2009; Provenzale et al., 2007). The paucity of studies spanning this developmental period reflects the challenges inherent in obtaining high quality data in healthy infants and toddlers using MRI, a notoriously motion-sensitive and noisy technique. However, as many psychiatric disorders are believed to have their genesis within this period (Hughes, 2007; Lewis and Levitt, 2002; Panzer and Viljoen, 2005; Zwaigenbaum et al., 2004), improved understanding of white matter development during this period is essential.

A more specific measure of myelin maturation may be provided by multi-component analysis of T_1_ and T_2_ relaxation, termed multi-component relaxometry (Kroeker and Henkelman, 1986; Menon et al., 1991; Whittall et al., 1997). Within brain parenchyma, T_2_ decay analysis reveals at least two discrete micro-anatomical water domains; a fast-relaxing water pool that is commonly associated with the water bound within the lipid bilayers of the myelin sheath, and a slower-relaxing water pool corresponding to water in and outside the myelinated axon (MacKay et al., 2006; Whittall et al., 1997). By quantifying the myelin-bound water signal, termed the myelin water fraction (MWF), a surrogate measure of myelin content is obtained that correlates strongly with histological assessments (Gareau et al., 2000; Laule et al., 2006; Webb et al., 2003). In addition, MWF has been shown to have greater myelin-specificity than diffusion anisotropy or magnetization transfer imaging (Madler et al., 2008; Vavasour et al., 2011). Deoni et al. (2011) have further demonstrated the application of multi-component relaxometry to the study of infant brain development, showing qualitative agreement between MR-derived MWF trends and the histologically-determined spatio-temporal patterns of myelination.

In this work, we aimed to further investigate the utility of MWF imaging in the study of white matter and myelin development throughout early childhood (defined herein as from 3 months through 5 years of age) and to compare MWF trends with more commonly acquired T_1_ and T_2_ relaxation time measures over this developmental period. Optimized, age-specific acquisition protocols were developed for non-sedated natural sleep imaging, permitting an overall success rate in excess of 95%. Developmental trajectories of MWF, T_1_ and T_2_ were obtained for frontal, temporal, occipital and parietal white matter regions, as well as for the genu, splenium and body of the corpus callosum. Correlations between MWF, T_1_ and T_2_ suggest that these measures are sensitive to differential aspects of microstructural development, with T_2_ consistently plateauing before T_1_ and MWF; and T_1_ and T_2_ having inconsistent (positive and negative) relationships with MWF over different developmental periods. Our results show that 1) albeit challenging, high quality MR imaging can be successfully and routinely performed within non-sedated infants, toddlers and young children; 2) derived spatio-temporal of MWF changes is in good agreement with histological studies of myelin maturation; and 3) care should be taken in linking relaxation time changes with myelin changes.

## Methods

### MWF imaging with mcDESPOT

**Fig. 1:**
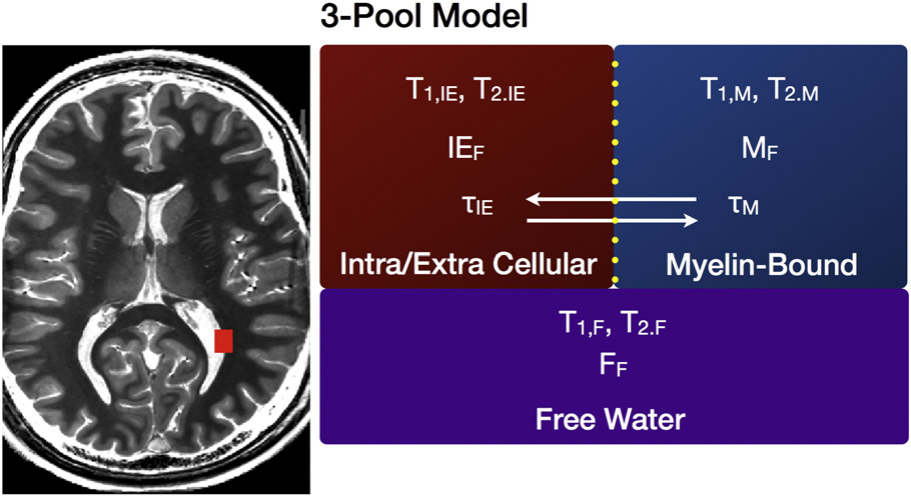
Graphical description of the three-pool tissue model used to fit the mcDESPOT data. The model comprises two exchange water pools (intra/extra cellular or axonal water, and the myelin-bound water) and a third non-exchange free water pool (broadly corresponding to cerebral spinal fluid).

Traditionally, multi-component relaxation imaging utilizes a multi-echo spin-echo (MESE) T_2_ approach (Whittall et al., 1997) that, unfortunately requires impractically long acquisition times (up to 15 min for 8–12 contiguous image slices) for routine applications. To acquire whole-brain, higher spatial resolution (1.8 × 1.8 × 1.8 mm^3^ voxel dimensions) MWF maps, we utilized a rapid alternative to MESE, termed mcDESPOT (multi-component driven equilibrium single pulse observation of T_1_ and T_2_, Deoni et al., 2008), which derives the MWF from spoiled gradient echo (SPGR, spoiled FLASH) and fully-balanced steady-state free precession (bSSFP) data acquired over a range of flip angles. Additional inversion-prepared (IR)-SPGR acquisitions can be used to correct for transmit (B_1_) magnetic field inhomogeneities, and repeating the bSSFP data with different radio-frequency (RF) phase increments allows correction for main (B_0_) field variations (Deoni, 2011). MWF maps are calculated by fitting the SPGR and bSSFP data to a three-pool model (illustrated in Fig. 1) that comprises two exchanging water pools (the myelin water and water inside and outside the axon) and a non-exchanging ‘free’ water pool (Deoni, 2012). This is unlike conventional T_2_-based approaches (Whittall et al., 1997) that derive MWF solely from T_2_ decay data with a less complicated (non-exchanging) model. As mcDESPOT is an extension of the single-component T_1_ and T_2_ estimation methods, DESPOT1 and DESPOT2, these values can also be estimated.

An acoustically-modified version of mcDESPOT, previously and employed in an earlier study of infant neurodevelopment (Deoni et al., 2011) was used in this study. To minimize acoustic levels (to less than 80 dB), the maximum gradient amplitude and slew rate were reduced, at the expense of increased scan time. Increased scan time also reduces energy deposition and specific absorption rate (SAR), an important consideration in pediatric imaging.

### Pediatric mcDESPOT imaging protocols

Due to the rapid growth of the infant brain, a single imaging protocol spanning 3 months through 5 years of age may not be the best approach. This may lead to overly lengthy scan times in the youngest children or sacrificed resolution in the eldest. Thus optimized protocols may afford the best tradeoff between these extremes.

As reported by others (Courchesne et al., 2011; Lenroot et al., 2007), subject motion in the youngest participants (under 4 years) is best minimized through restful scanning. Participants older than 4 years of age can usually remain still for short time periods provided they are suitably entertained (watching a movie, TV show, etc.).

Thus, we developed five age-specific mcDESPOT protocols (outlined in Appendix A), which met the following five criteria: 1. Whole-brain acquisition; 2. Consistent spatial resolution; 3. Noise levels less than 60 dB for the youngest infants to allow scanning during sleep, and less than 90 dB for older participants; 4. Total imaging time less than 25 min; and 5. Optimization of acquisition parameters to maximize MWF precision.

Field-of-view (FOV) was informed by mean head circumference (www.cdc.gov/growthcharts/clinical_charts.htm) and image matrix size was varied to provide a consistent (1.8 × 1.8 × 1.8) mm^3^ isotropic voxel volume. Scan time was kept below a tolerable limit of 25 min, varying from 18:22 to 24:20. Unprotected acoustic levels were below 85 dB (and less than 60 dB for the youngest participants). Additional acoustic noise reductions were achieved through passive measures, including a sound-insulating bore insert (Quiet Barrier HD Composite, UltraBarrier USA), MiniMuff noise attenuators (Natus, USA), and electrodynamic headphones (MR Confon, Germany). Acquisition parameters were optimized through consideration of the mean white matter relaxation times at each age (Saito et al., 2009) as described previously (Deoni, 2007, 2011).

### Imaging myelination in healthy infants, toddlers and young children

153 healthy infants (67 female) born at term and between the approximate ages of 3 months to 5.5 years (76 to 2040 days corrected to a 40-week gestation) were imaged using their age-appropriate protocol on a Siemens Tim Trio scanner with an 8-channel head RF array. Brief gender/age information is provided in Table 1. Informed consent was obtained from each participating family, and the study performed with approval from the local institutional review board.

Inclusion criteria for the study were: 1. Uncomplicated single birth between 37 and 42 weeks (all ages were corrected to a 40 week gestation); 2. No exposure to alcohol or illicit drugs during pregnancy; 3. No familial history of major psychiatric or depressive illness; 4. No diagnosis of major psychiatric, depressive or learning disorder in participant; 5. No pre-existing neurological conditions or major head trauma; and 6. No abnormalities detected on fetal ultrasound.

Infants and toddlers under 48 months (1440 days) of age were imaged during non-sedated sleep. Young children older than 48 months were imaged while watching a favorite movie or TV show. For all children, an infant or child-sized MedVac vacuum immobilization bag (CFI Medical Solutions, USA) and foam padding were used to minimize intra-scan motion. Infants were continuously monitored in the darkened scanner suite using a pediatric pulse-oximetry system and infrared camera. A research nurse and research assistant, and parents if they chose, were present in the scanner room to visually monitor each participant.

Following image acquisition, each participant's individual SPGR, IR-SPGR and bSSFP images were linearly co-registered to account for any subtle head motion; non-parenchyma signal removed; and the MWF estimated at each brain voxel (Deoni, 2012) to create MWF maps for each infant. Voxel-wise single-component T_1_ and T_2_ maps were also estimated.

### Alignment of infant and toddler myelin fraction maps and development of age-specific templates

**Fig. 2:**
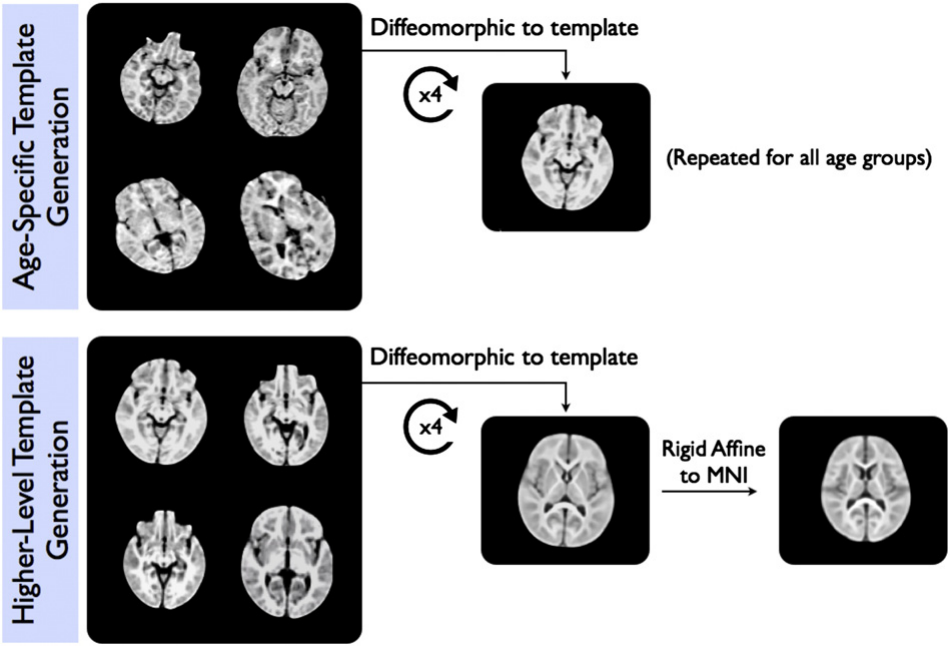
Illustration of the process used to calculate the age-specific and overall study-specific T_1_-weighted image templates.

Following calculation of each participant's MWF map, each was non-linearly aligned to a study specific T_1_-weighted template in approximate MNI space. This registration was performed using the high flip angle SPGR images (with transformations applied to the quantitative MWF, T_1_ and T_2_ maps retrospectively) as follows (and illustrated in Fig. 2).

1. Participants were divided into 12 age-groups (3, 6, 9, 12, 15, 18, 21, 24, 30, 36, 48 and 60 months). Where possible, 8 subjects were randomly selected within each group (except for the 18 and 21 month groups, were only 7 and 6 subjects were available, respectively).
2. For each age group, a T_1_-weighted template was created using symmetric diffeomorphic normalization (SyN; Avants et al., 2008) as implemented in the ANTs package and a cross-correlation similarity measure (http://picsl.upenn.edu/ANTS). This was performed using the buildtemplateparallel.sh script distributed with the ANTs package (Avants et al., 2010).
3. A further, higher-level, template was created from the underlying age-specific templates and a rigid affine transformation was calculated from the space of this higher-level template to the MNI T_1_ template.
4. The 36 and 48 month age groups were refined to 36, 42, 48 and 54 months (with the 36 month template calculated in (2) used for the 36 and 42 month groups; and the 48 month template calculated in (2) used for the 48 and 54 month groups). These were not treated separately initially due to the small numbers in each.
5. For each participant, the high-angle SPGR image was non-linearly aligned to their respective age-specific template, calculated in (2). The quantitative MWF, T_1_ and T_2_ maps were transformed into the 1.5 mm^3^ space of the approximate MNI template using these transformations and those calculated in (3).

**Fig. 3:**
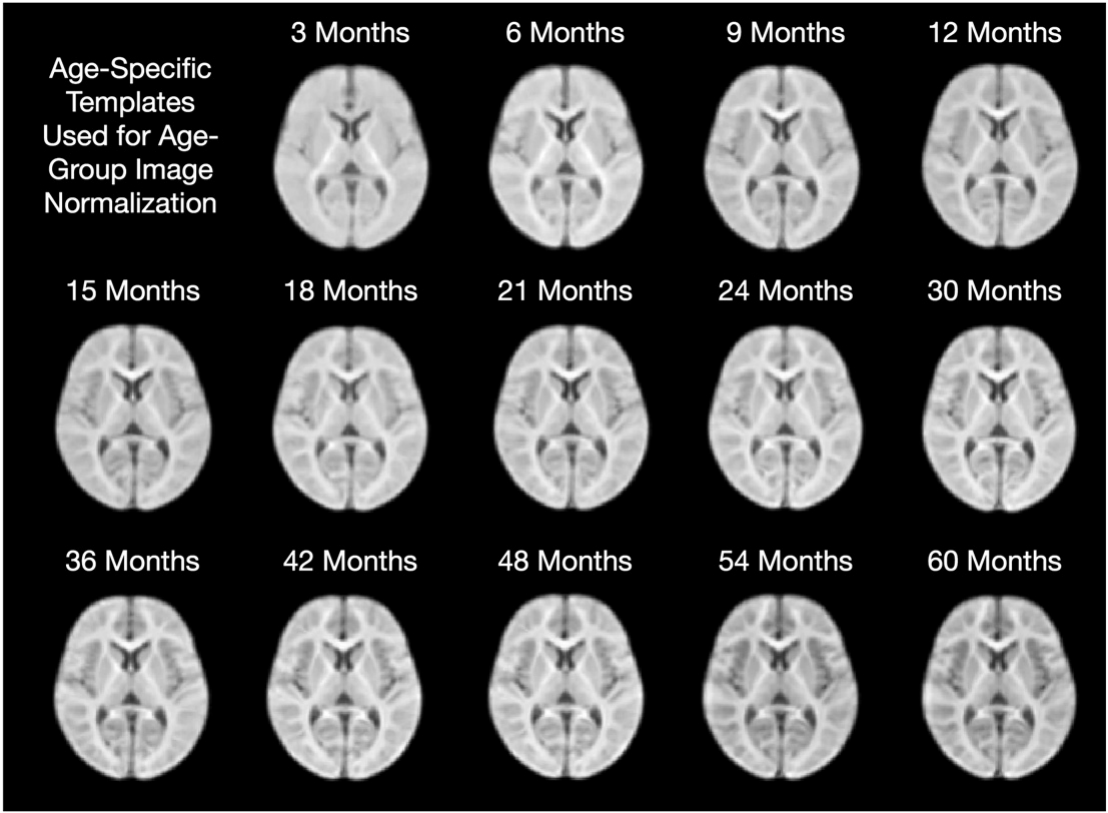
Matched axially-oriented slices from each of the age templates in approximate MNI space.

Representative matched axial slices through each of the age templates are shown in Fig. 3.

### Investigation of regional MWF, T_1_ and T_2_ trajectories

With all data spatially aligned in a similar analysis space, mean developmental MWF, T_1_ and T_2_ trajectories were obtained for the genu, splenium and body of the corpus callosum, right and left hemisphere cingulum, corona radiata, internal capsule and optic radiation, and right and left hemisphere frontal, occipital, temporal, parietal and cerebellar white matter regions.

Anatomical masks for each of these regions (derived as outlined below) were superimposed onto each infant's dataset, and the mean and standard deviation calculated for each region. Only voxels with MWF values greater than 0.001 were included in the regional means.

For the frontal, occipital, parietal, temporal and cerebellar white matter,

1. A global binary white matter mask was calculated by thresholding the MNI white matter probability image provided within FSL (www.fmrib.ox.ac.uk/fsl) at 180.
2. Masks of the frontal, occipital, parietal, temporal and cerebellar lobes were obtained from the MNI database (Mazziotta et al., 2001). These were multiplied by the binary global mask (1) to obtain the regional white matter masks.
3. The white matter masks for each region were divided by hemisphere.
4. The registration transformation between the MNI template and the study template was calculated, and each masked transformed to the study space.

**Fig. 4:**
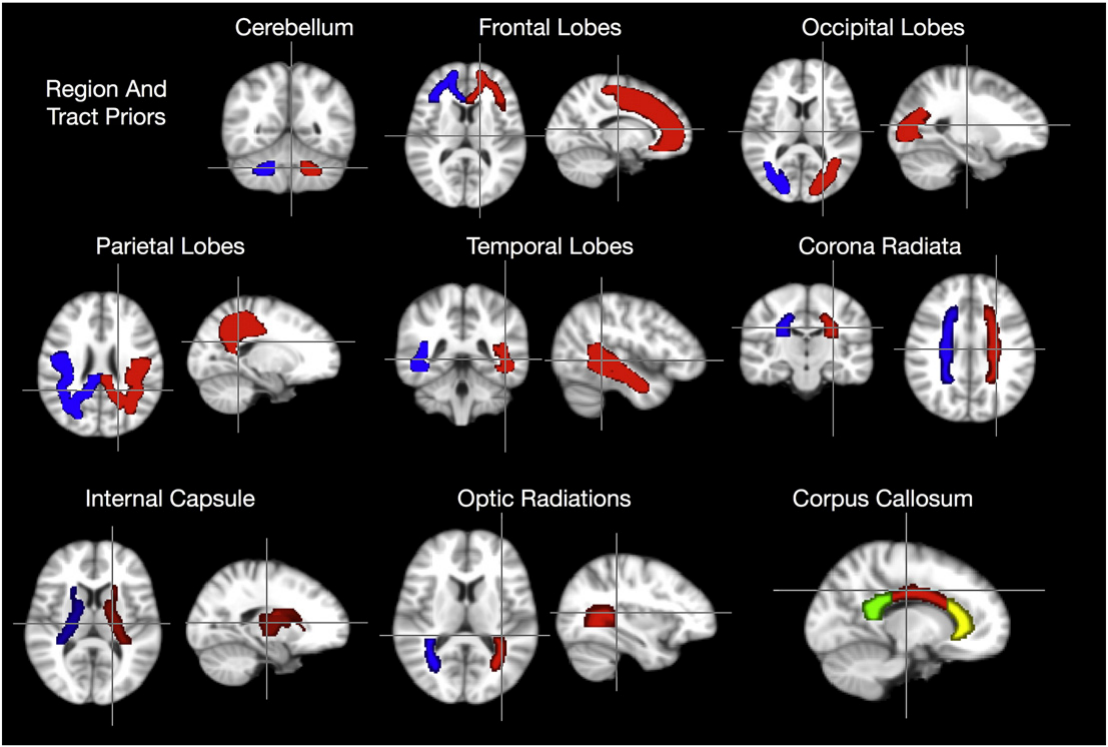
Brain region and tract overlays used for regional analysis and comparisons. Red = right, blue = left.

For the white matter tract masks, including genu, body and splenium of the corpus callosum, cingulum, corona radiata, internal capsule, and optic radiation, this same process was applied to the John Hopkins University DT-MRI white matter atlas (Oishi et al., 2008). The corona radiata mask comprised the anterior, superior and posterior portions; the internal capsule mask comprised the anterior, posterior and retrolenticular portions. Each of these regions, superimposed onto the mean study template is shown in Fig. 4.

Pearson correlations between MWF and T_1_; and MWF and T_2_ were calculated for each white matter tract and region across the full age range; as well as across developmental periods between 1) 0 and 6 months of age; 2) 6–12 months; 3) 12–24 months; 4) 24–36 months; 5) 36–48 months; and 6) 48–60 months of age.

### Modeling MWF trajectories

Using the mean MWF values obtained for each brain anatomical and tract region, logarithmic curve modeling was used to delineate potential hemispheric and gender differences in development. Although logarithmic curves do not fully capture the earliest maturational dynamics (that is, MWF starts at, or close to, 0 exponentially increases over the first 12 to 18 months, followed by slower growth throughout the rest of childhood, Fig. 6), they do, however, capture the overall temporal changes.

Logarithmic curves of the form *MWF*(*age*) = *α*ln(*age*) − *β* were fit to the mean data from each brain region and tract. To determine if hemispheric differences were present, curves were fit to data from each hemisphere independently (dual-curve model), as well as to the combined data (single-curve model). An F-test was then used to determine whether the dual-curve model was justified. Similarly, curves were fit to the data obtained from each gender independently as well as pooled, and an F-test used to determine if gender differences were present.

## Results

153 successful datasets were obtained from 159 infants who underwent scanning, representing a successful scanning rate of approx. 96%. However, not all children were placed in the scanner on the first visit. 52 infants and toddlers (between the ages of 16 and 60 months) either failed to fall asleep on their first visit or were hesitant about going in the scanner. Of these, 28 chose not to return. The other 24 returned for a second visit and all were successfully scanned. These rates compare favorably to other infant imaging studies (Courchesne et al., 2011; Dubois et al., 2006; Lenroot and Giedd, 2006), and show that imaging across this challenging age-range is possible with appropriate imaging sequences and research team. Time required for each visit (from arrival, falling asleep and scanning) ranged from less than 45 min to more than 3 h.

**Fig. 5:**
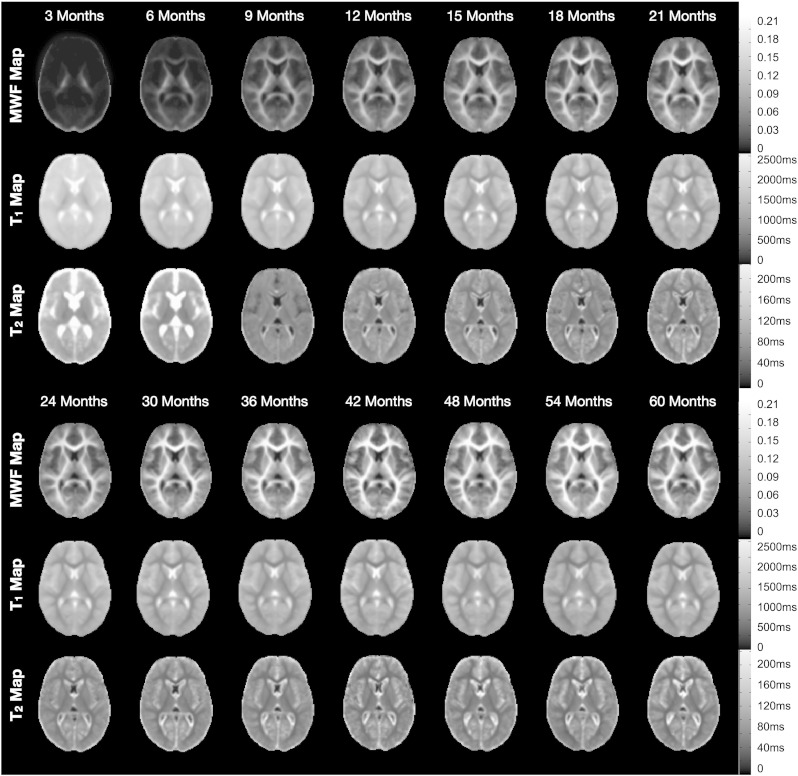
Matched axially-oriented slices through the mean MWF, T_1_ and T_2_ maps from each age-group. For the 3 and 6 month data, T_2_ values were calculated for all voxels. For 9 months and above, T_2_ was only calculated in voxels with a corresponding T_1_ less than 3500 ms.

**Fig. 6:**
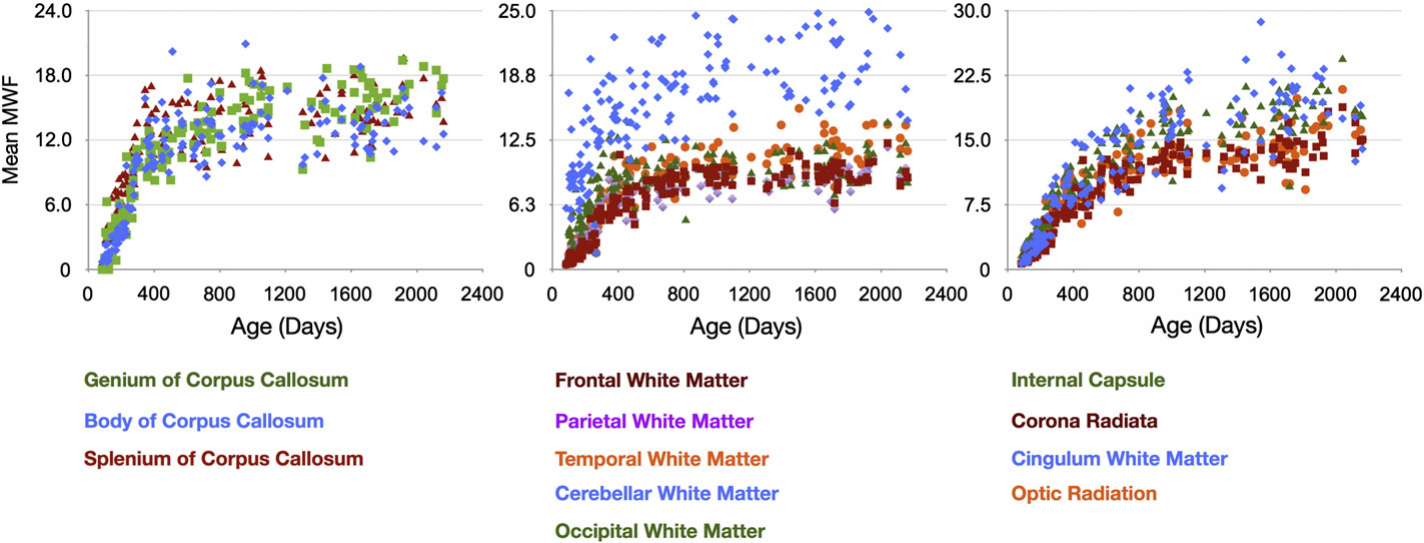
Gender-combined myelination trajectories for each white matter region and pathway spanning 83 through 2040 days of age. Points represent the mean values with error bars corresponding to the measurement standard deviation.

Corresponding matched axial slices through the MWF, T_1_ and T_2_ maps of a representative infant from each age-group are shown in Fig. 5. For data under 9 months, T_2_ values were calculated for each imaging voxel, however, beyond 9 months T_2_ was only calculated for voxels with T_1_ values less than 3500 ms. This differential processing is the reason behind the differing presentation of the 3 and 6 month mean T_2_ maps (showing values within the ventricles and surrounding cerebral spinal fluid) and the remaining T_2_ maps in which these areas appear masked. The MWF data illustrate the progressive advancement of myelination throughout the brain. The spatio-temporal pattern of myelination demonstrated by these data closely resembles post-mortem studies (Yakovlev and Lecours, 1967). We observe myelination beginning in the cerebellum and internal capsule prior to 3 months. Myelination then proceeds to the splenium of the corpus callosum and optic radiations; the occipital and parietal lobes and body of the corpus callosum; and genu of the corpus callosum. The last regions to myelinate, as predicted by histology, are the frontal and temporal lobes. Analogous trends of maturation are seen in the quantitative T_1_ and T_2_ data, which also show progressive reductions in white and gray matter values across the age span.

**Fig. 7:**
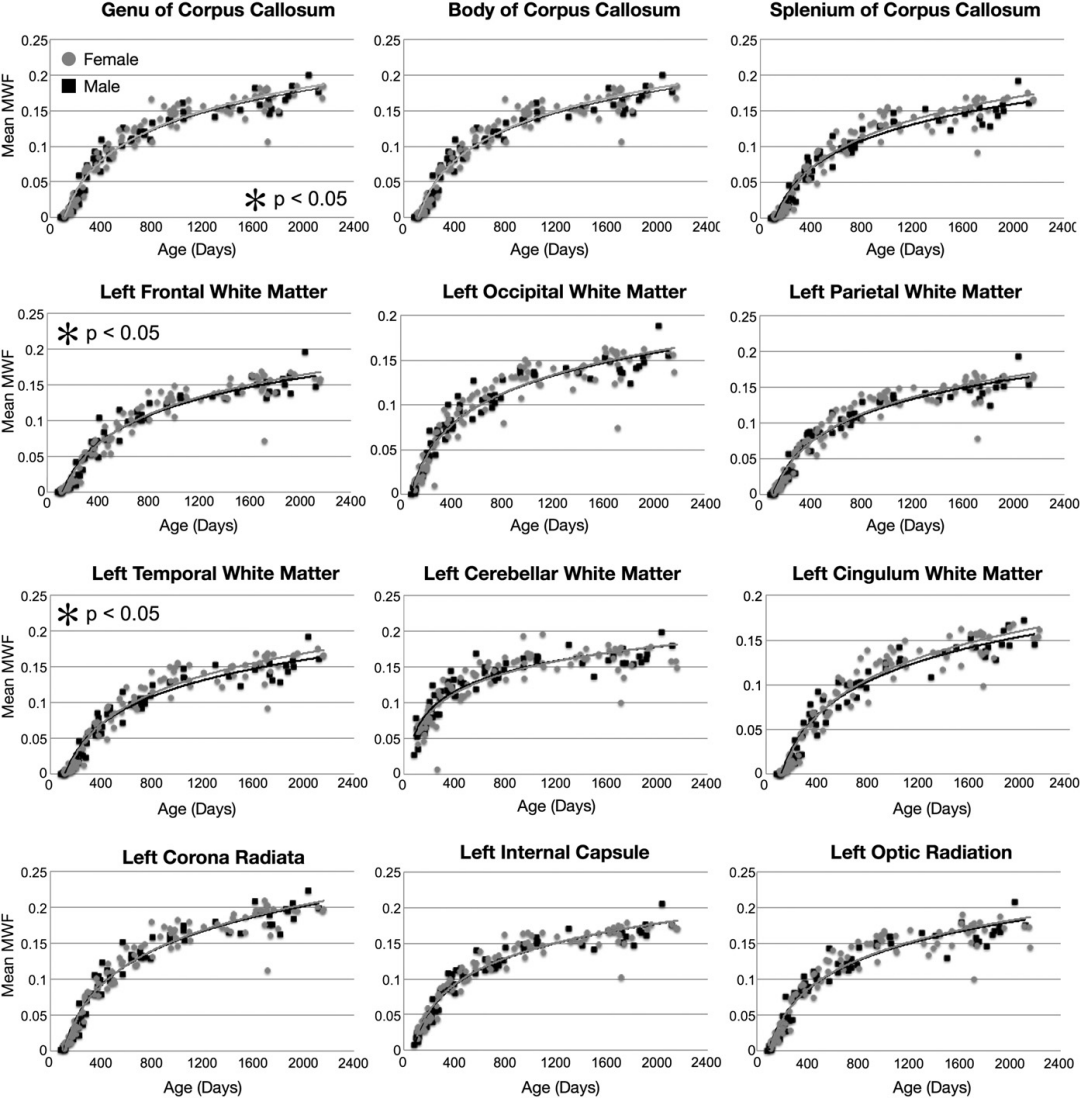
Myelination trajectories, separated by gender, for each left hemisphere and midline white matter region and pathway spanning 83 through 2040 days of age. Points represent the mean value obtained from each region. Female values are denoted by gray circles. Plots marked with an asterisk correspond to those regions were a significant (p < 0.05) male–female difference was found.

**Fig. 8:**
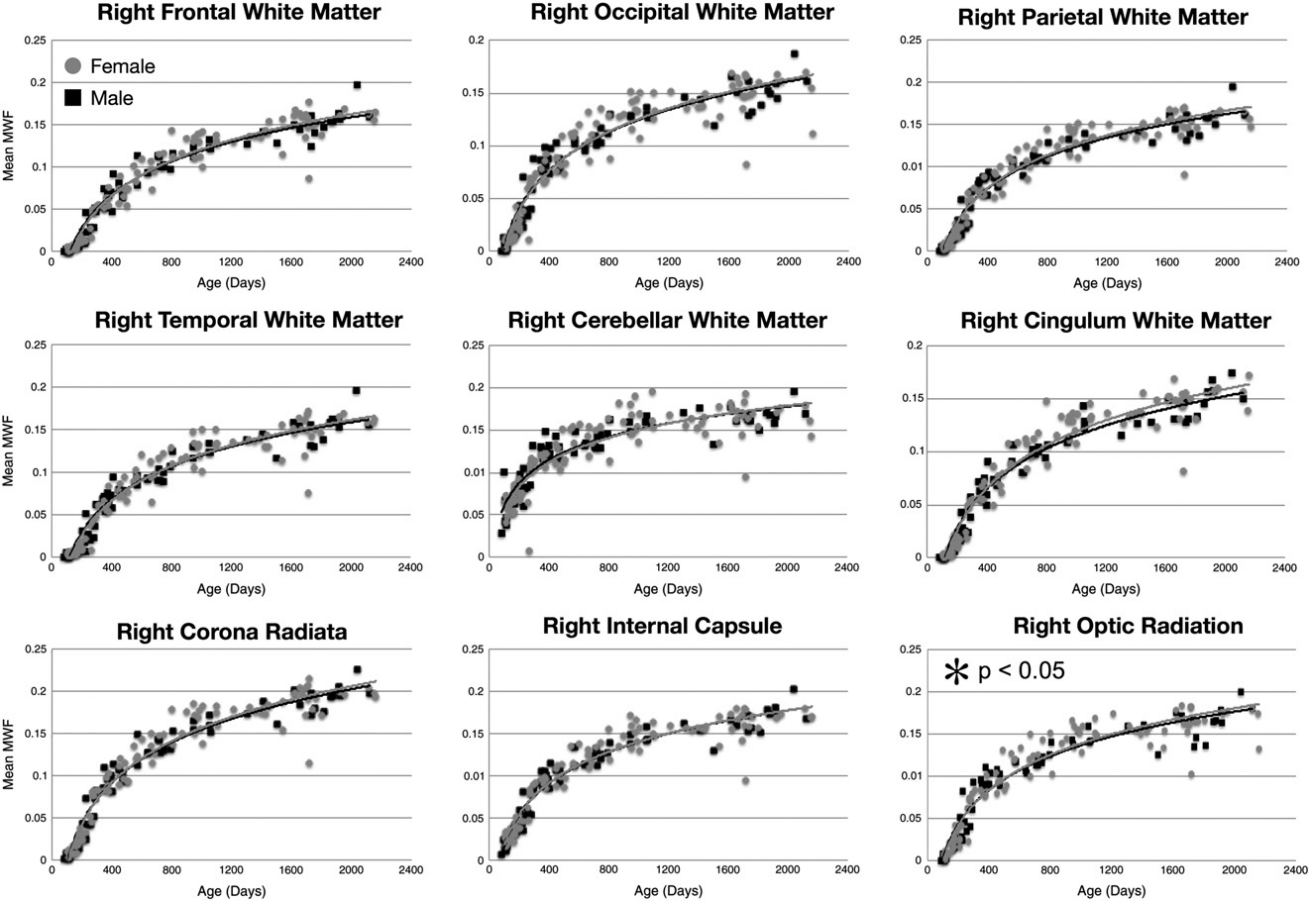
Myelination trajectories, separated by gender, for each right hemisphere white matter region and pathway spanning 83 through 2040 days of age. Points represent the mean value obtained from each region. Female values are denoted by gray circles. Plots marked with an asterisk correspond to those regions were a significant (p < 0.05) male–female difference was found.

Mean male and female MWF trajectories for each of the investigated white matter regions are shown in Figs. 7 and 8. Superimposed on the mean data are the logarithmic curves calculated for each genders data. Using an F-test to discriminate regions with gender differences, we found significant (p < 0.05 corrected for multiple comparisons) male/female growth differences in the genu of the corpus callosum, left frontal white matter, left temporal white matter and right optic radiation. In each case, females showed an increased developmental rate compared to males. A summary of the fit curve equations and F-stat values is shown in Table 6. No hemispheric differences in growth rate were found (Table 7).

Overall, the myelination trajectories follow a sigmoidal shape, with a lag period followed by exponential growth over the first 12–16 months and slower growth from 2 through 5 years of age. By 60 months, the white matter regions are approaching myelin water fraction values close to 0.2, approx. 80% of values measured in adult white matter (Deoni et al., 2011). Previous studies of white matter development (Bartzokis et al., 2010; Cho et al., 1997) show white matter and overall brain growth continues into the second and, in some brain regions, third decade of life.

**Fig. 9:**
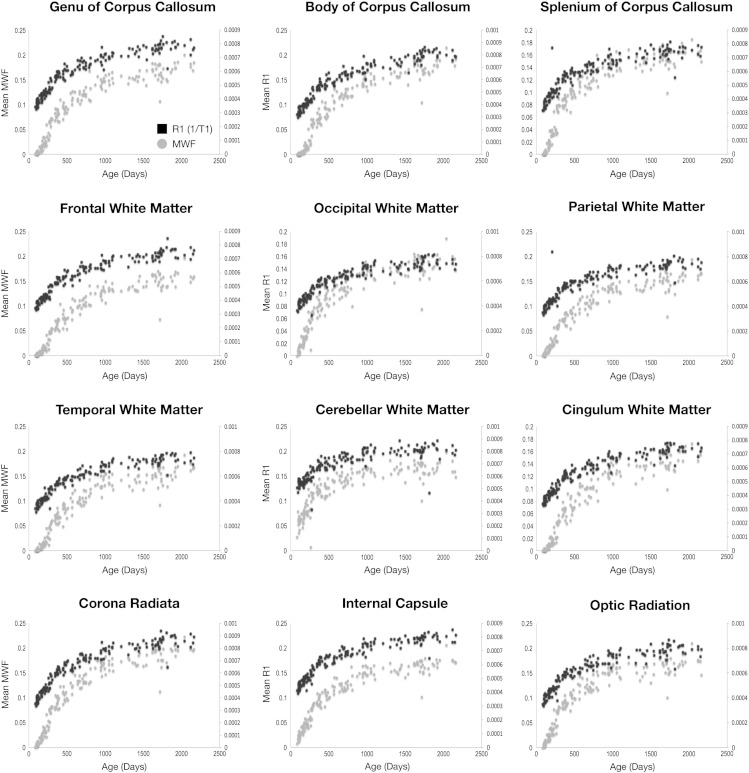
Comparison of MWF (gray circles) and R1 (1/T_1_) (black squares) trajectories for each white matter region and pathway across the age-span.

**Fig. 10:**
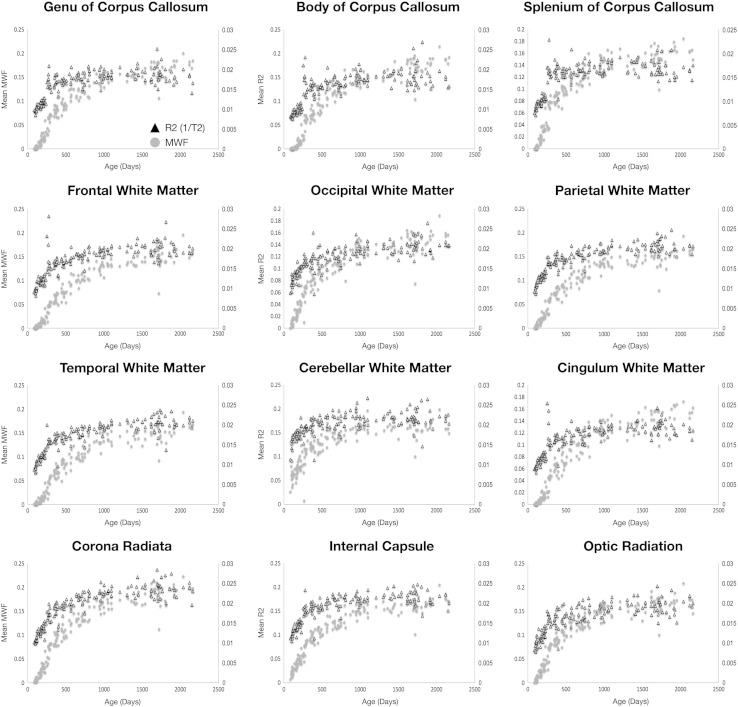
Comparison of MWF (gray circles) and R2 (1/T_2_) (black triangles) trajectories for each white matter region and pathway across the age-span.

Comparison of MWF and R1 (1/T_1_) vs. age curves for each region is shown in Fig. 9. MWF and R2 (1/T_2_) vs. age curves are shown in Fig. 10. Both R1 and R2 follow approximately logarithmically shaped curves, whereas the MWF curves are sigmoidal. This reflects the differential sensitivity of these measures. Myelin is not histologically present at birth (except in the cerebellum and brainstem) and, thus, the MWF values are zero in all other brain regions. In contrast, as T_1_ and T_2_ reflect the presence of water, measurable values are always present.

The MWF and R1 curves appear to have similar shape and slope between 2 and 5 years of age, with the curves approximately parallel to each other. This may suggest that they reflect similar changes over this period. In contrast, the R2 curves quickly plateau at 2 years, showing only subtle increases beyond this age point. This contrasts with the MWF curves, which continue increasing throughout childhood.

To examine the associations between MWF, T_1_ (R1) and T_2_ (R2) more quantitatively, Pearson R values were calculated (and converted to T statistics) across the full age range; as well as across more defined developmental periods (0 and 6 months of age; 6–12 months;12–24 months; 24–36 months; 36–48 months; 48–60 months). A summary of these results is provided in Tables 2 and 3. Statistical significance was defined at p < 0.05 (uncorrected for multiple comparisons). When all data was included, statistically significant correlations between MWF and R1 were found in all regions except left occipital white matter and right optic radiation. Between MWF and R2, statistically significant correlations were found in all regions except the left cerebellum.

When the relationships between MWF, R1 and R2 were investigated over more discrete age periods, a less coherent picture is noted. For example, focusing on R2 measures, between 3 and 6 months, MWF and R2 are well correlated in all investigated regions except the right cerebellum. Between 6 and 12 months, only the corpus callosum, bilateral internal capsule and corona radiata, and right optic radiation and right cingulum show significant correlation. Between 12 and 24 months, the body and genu of the corpus callosum, bilateral frontal, cingulum, corona radiata and left internal capsule and temporal white matter show significant correlation. By 36 months, there is no correlation between MWF and R2.

Investigating R1 vs. MWF, we note different regions with significant correlations than observed with R2. For example, under 6 months, body and splenium of the corpus callosum, bilateral cingulum, optic radiation, corona radiata, parietal white matter, and left frontal and internal capsule are significantly correlated with MWF. Between 6 and 12 months, values within the optic radiations, parietal white or splenium of the corpus callosum are no longer correlated, but the genu, temporal white matter and bilateral internal capsule are. As with R2, by 36 months few areas show significant correlations between R1 and MWF, and those that do are surprisingly negative correlations (increased MWF corresponds to decreased R1—or increased T_1_).

Investigating these relationships further, we also sought to determine the direct relationships between MWF and R1 and R2, accounting for the effect of age. Age-corrected partial correlations (Pearson R) were calculated for the defined developmental periods (0 and 6 months of age; 6–12 months;12–24 months; 24–36 months; 36–48 months; 48–60 months). These age groups were chosen because the data could be approximated as linear within these regions. Results of this analysis are shown in Tables 4 and 5, with statistical significance defined at p < 0.05 (uncorrected for multiple comparisons). These results yield sporadic associations, mainly between MWF and R1 between 12 and 24 months of age, suggesting that MWF is a complementary, but distinct, measure of maturation from relaxometry values.

Cumulatively, the results shown demonstrate the ability to reliably acquire high quality data over the early childhood age-range. Though optimized imaging protocols were used for each age group, the signal-to-noise ratio (SNR) of the calculated MWF maps (myelin water fraction to noise ratio, MWFNR) was not consistent across the age-spectrum, ranging from a low of 7 at 6 months of age, to a high of 20 by 2 years of age (and remaining at this value throughout the rest of the age range). This value was obtained by calculating the mean MWF value/standard deviation for each of the brain regions investigated, and the averaging across the regions. This variable MWFNR may have future implications for modeling, necessitating a weighted least squares approach, as well as in the ability to accurately discriminate subtle MWF differences at early ages. Of note, however, quality and cross-sectional agreement of the data contradict a recent theoretical analysis of mcDESPOT (Lamkford and Does, 2012), which cautioned that the method was incapable of producing reproducible results. The qualitative agreement with prior histologically-determined patterns of myelination and white matter development further underscore the ability of mcDESPOT to provide salient information related to myelin content through the quantification of myelin water fraction. It is, however, possible that mcDESPOT is influenced by additional effects, such as magnetization transfer.

## Discussion

In this work, we have investigated the utility of MWF imaging to investigate white matter development across the childhood period from 3 months through 5 years of age. From a cohort of 153 healthy male and female children, we have obtained regional myelination trajectories that exhibit growth-rate differences corresponding to prior histologically-defined timelines. We have further performed the first comparison of MWF values with T_1_ and T_2_ values across this period, revealing that while intermittently correlated, these techniques provide complementary but differential information.

Using mean regional values, we investigated both hemispheric and gender differences in development. An overlay of the investigated regions is shown in Fig. 4. It should be noted that while the regions show good anatomical localization, all masks were derived from adult templates. While this may not be problematic for the white matter regions (frontal, temporal, occipital, parietal, and cerebellar white matter), differences in tract-size and extent between infants and adults may have resulted in our overestimating the size of different tracts. The use of a pediatric-centric template (for example, that of Hermoye et al., 2006) may serve to alleviate these concerns. Similarly, pediatric derived anatomical atlases are also now becoming more readily available (Fonov et al., 2011), which may increase anatomical specificity. This is particularly true for the youngest infants were, presumably, tracts and region areas would be most over-estimated, leading to artificially downwardly biased estimates of regional mean MWF. However, since mean MWF values were calculated only from voxels with a MWF greater than 0.001, it is not anticipated that larger region masks would significantly bias mean values obtained from the youngest infants.

Based on this regional analysis, we anticipated finding hemispheric differences in myelination, particularly within the temporal and frontal regions due to functional lateralization (Erberich et al., 2006), however, significant differences, defined by a single vs dual-curve F-test, were not observed. This could reflect a low sensitivity of mcDESPOT to maturational change, with such differences being too small for direct discrimination, or differences being constrained to more anatomically eloquent areas than the gross regions investigated. Alternatively, this lack of finding could be in part due to the fitting of logarithmic curves to data that is more sigmoidal in shape in the earliest stages of development. However, the lack of hemispheric asymmetry agrees with prior studies of white matter volume development in young children (Durston et al., 2001).

As almost all neuropsychological disorders have differing prevalence and/or presentation in males and females, it was also hypothesized that gender differences would be apparent in the MWF trajectories. Studies of cortical thickness, as well as white and gray matter volume, consistently demonstrate sexual dimorphism (Giedd et al., 1996; Lenroot and Giedd, 2010; Lenroot et al., 2007). From our data, gender-based differences in development were detected in some brain regions (genu of the corpus callosum, left frontal and temporal white matter, and right optic radiations). In each region, girls were shown to increase more rapidly than boys, though with little MWF volume difference at 5 years of age. These results are in general agreement with prior literature (see Durston et al., 2001 for a thorough review) showing little male–female difference in white matter volume between brain regions. However, it should be noted that the majority of prior studies have focused on older children (4 years and older), with little data available between the ages of 1 and 4 years. The notable exception is the work of Courchesne et al. (2000) who, although not showing specific regional gender differences, showed an overall reduction in female white matter volume relative to males after 4 years of age. Although the relationship between MWF and white matter volume is not known, it is reasonable to assume that increased MWF is associated with increased tissue volume. Investigation of children beyond 5 years, therefore is warranted. Our results may also be compared against prior studies of fractional anisotropy (FA, a non-specific measure of white matter ‘integrity’). Increased FA in the left frontal lobe of women compared to men has been previously shown (Szeszko et al., 2003). While significant leftward FA asymmetry has been shown (Kang et al., 2011), this result comes from older males and females (mean age = 27 years) and may not reflect early differences.

It is important to note that hemisphere and gender differences were examined herein within the context of rate, and not extent. It is possible that group differences might exist that could be detected by grouping individuals of similar age and performing a voxel-by-voxel analysis of laterality (Dubois et al., 2009), or voxel-by-voxel *t*-test between genders. Such analysis may be more sensitive to differences, and more directly comparable to prior volumetric and diffusion tensor based studies.

A particularly striking observation is the inconsistent relationships noted between MWF and the more common T_1_ and T_2_ measures. The presented results, which show associations in different structures during different developmental periods, and interspersed positive and negative correlations, suggest that while these measures are complimentary, they do not inform on the same microstructural changes. This result has implications on studies that have used relaxometry measures (or T_1_ and T_2_-weighted signals) as surrogate markers of myelin content (Bartzokis et al., 2010; Glasser and Van Essen, 2011; Van Essen et al., 2012). It is well known that the relaxation properties of tissues are tightly coupled, but notoriously non-specific, to different biochemical and biophysical alterations (MacKay et al., 2006). For example, early brain maturation is accompanied not only by the establishment of the myelin sheath itself, but also by the arrival of precursory lipid and proteins, compartmentalization of water, and iron within the oligodendrocytes. Each of these processes can lead to changes in both T_1_ and T_2_. In particular, compartmentalization and the presence of large lipids and proteins have dramatic effects on T_1_, while iron content significantly influences T_2_. It, perhaps, should not be surprising that T_1_ and T_2_ decrease during early brain development, and they are not sensitive solely to the embellishment of the myelin sheath itself, which may be more sensitively detected through MWF. Based on the results shown herein, specifically the lack of significant age-corrected MWF vs T_1_ or T_2_ correlations, care should be taken in ascribing changes in T_1_ and T_2_ solely to myelin content change.

Beyond the analysis shown herein, the data lends itself to furthering our understanding of cortical development, specific white matter pathway maturation, and structure–function associations linking white matter development with evolving behavioral and cognitive functioning. As brain networks consist of both cortical and subcortical regions connected by white matter pathways, it is anticipated that maturation of the white matter pathways will be reflected in cortical maturation, and vise versa. Early work toward understanding network development has been demonstrated in the linguistic network of early infants (1–4 months) using cortical segmentation and diffusion tensor imaging (Leroy et al., 2011). The acquired data lends itself to similar analysis with the prospect of investigated gender differences in development, as well as over a wider developmental age window. Taking an additional step, network maturation could be further linked with cognitive development, for example in expressive and receptive language, using paired assessments using the Mullen Scales of Early Learning (Mullen, 1995) or other age-appropriate assessment battery.

## Conclusion

The development of myelinated white matter is an important aspect of neurodevelopment. In this work, we have outlined a series of imaging protocols designed to successfully investigate myelination in infants, toddlers and young children. The presented work compliments a growing body of maturational studies performed with T_1_ and T_2_ weighted imaging, DT-MRI and functional MRI. We have shown regional myelination trajectories across the spectrum of early childhood for the first time and have demonstrated the differential information provided by MWF mapping across this age-group.

## Figures and Tables

**Table 1:** Male/female age information for 153 scanned participants. Ages are given in GC days.

	3 months	6 months	9 months	12 months	15 months	18 months	21 months	24 months	30 months	36 months	42 months	48 months	54 months	60 months
Male	6	13	8	6	6	4	1	5	10	7	2	5	5	15
Female	7	7	6	6	3	3	5	3	3	2	1	2	7	5
Totals	13	20	14	12	9	7	6	8	13	9	3	7	12	20
Age (min/max)	76/125	137/224	230/329	344/403	410/489	500/583	587/680	689/763	791/981	994/1118	1210/1317	1354/1501	1541/1709	1716/2040
Age (mean)	105.5	180	278	372	458	548	634	727	905	1058	1264	1409	1643	1778

**Table 2:** Summary of associations (T-statistics) between MWF and R1 (1/T_1_) over different brain white matter regions and pathways. Bold type denotes significant (p < 0.05, uncorrected) correlations.

R1							
Region/tract	0–6 months (N = 25; critical T = 2.06)	6–12 months (N = 26; critical T = 2.06)	12–24 months (N = 35; critical T = 2.03)	24–36 months (N = 23; critical T = 2.06)	36–48 months (N = 11; critical T = 2.20)	48–60 months (N = 35; critical T = 2.03)	Overall (N = 153; critical T = 1.98)
Body (corpus callosum)	**2.38**	**5.27**	1.79	1.46	0.50	− 0.81	**32.6**
Genu (corpus callosum)	1.83	**4.87**	**2.67**	1.24	1.52	− 2.06	**32.8**
Splenium (corpus callosum)	**2.96**	1.54	**2.45**	1.89	0.86	− 0.94	**26.2**
Left cerebellar white matter	− 0.08	1.13	**2.53**	0.89	− 1.00	− 0.03	**16.6**
Right cerebellar white matter	− 0.00	1.98	0.40	0.07	− 0.70	− 0.05	**16.8**
Left frontal white matter	**2.38**	**5.13**	1.65	**2.15**	− 0.02	− 1.81	**31.4**
Right frontal white matter	1.82	**5.47**	1.36	1.67	1.95	− 0.13	**13.5**
Left parietal white matter	**2.32**	1.51	**2.43**	**2.22**	− 0.76	− 0.38	**25.5**
Right parietal white matter	**2.66**	0.51	1.54	**2.52**	− 0.08	− 0.70	**17.5**
Left occipital white matter	1.74	− 0.32	**2.37**	0.83	**− 2.72**	− 0.98	− 0.0
Right occipital white matter	1.66	− 0.13	1.85	1.28	− 1.53	− 0.87	**2.7**
Left temporal white matter	1.59	**4.73**	**2.47**	**2.11**	**− 2.99**	0.06	**33.6**
Right temporal white matter	1.48	**2.65**	1.67	**2.81**	− 1.19	− 0.19	**27.8**
Left cingulum	**2.19**	**4.76**	1.58	1.20	0.96	− 1.05	**30.1**
Right cingulum	**2.41**	**4.36**	1.57	1.05	1.31	− 2.31	**30.1**
Left internal capsule	**2.55**	**4.53**	1.18	1.65	− 2.17	− 0.20	**30.8**
Right internal capsule	1.72	**5.06**	0.58	1.85	− 2.03	− 0.07	**28.5**
Left optic radiation	**2.67**	1.18	**3.42**	1.47	− 2.74	− 1.22	**19.9**
Right optic radiation	**2.49**	− 0.17	1.87	1.96	− 1.13	− 0.24	− 0.2
Left corona radiata	**3.13**	**5.57**	**2.28**	**2.11**	− 1.67	− 0.31	**34.4**
Right corona radiata	**2.47**	**5.74**	**2.22**	1.46	− 0.55	− 1.27	**33.7**

**Table 3:** Summary of associations (T-statistics) between MWF and R2 (1/T_2_) over different brain white matter regions and pathways. Bold type denotes significant (p < 0.05, uncorrected) correlations.

R2							
Region/tract	0–6 months (N = 25; critical T = 2.06)	6–12 months (N = 26; critical T = 2.06)	12–24 months (N = 35; critical T = 2.03)	24–36 months (N = 24; critical T = 2.06)	36–48 months (N = 11; critical T = 2.20)	48–60 months (N = 21; critical T = 2.08)	Overall (N = 141; critical T = 1.98)
Body (corpus callosum)	**3.53**	**3.37**	**2.67**	0.32	− 0.25	− 1.57	**17.9**
Genu (corpus callosum)	**3.62**	**2.47**	**3.92**	1.07	0.07	− 2.59	**14.8**
Splenium (corpus callosum)	**2.44**	**2.74**	1.24	0.81	− 0.13	− 0.83	**15.8**
Left cerebellar white matter	**2.14**	− 0.31	1.24	0.47	0.55	− 0.80	1.3
Right cerebellar white matter	0.77	− 0.07	0.86	0.96	0.26	− 0.71	**4.3**
Left frontal white matter	**3.42**	1.76	4.30	0.93	0.47	− 1.77	**15.7**
Right frontal white matter	**2.54**	1.04	**2.51**	**2.31**	0.18	− 0.78	**11.7**
Left parietal white matter	**4.22**	0.86	**2.63**	1.79	− 0.34	− 1.29	**15.7**
Right parietal white matter	**4.42**	1.84	1.21	1.40	1.44	− 0.51	**19.5**
Left occipital white matter	**3.01**	− 0.33	− 0.43	1.36	− 0.08	− 1.33	**3.1**
Right occipital white matter	**2.95**	0.47	0.61	0.06	0.13	0.33	**11.5**
Left temporal white matter	**4.01**	− 0.13	**2.81**	1.19	0.76	− 0.51	**5.3**
Right temporal white matter	**3.34**	− 0.05	1.57	**3.15**	1.14	0.18	**9.5**
Left cingulum	**3.88**	0.54	**2.86**	0.71	− 0.07	− 1.48	**10.1**
Right cingulum	**3.57**	**2.35**	**2.89**	0.48	1.21	− 2.10	**18.1**
Left internal capsule	**3.76**	**3.74**	**3.03**	0.69	− 0.45	− 1.00	**20.4**
Right internal capsule	**2.79**	**4.27**	1.39	**2.49**	− 0.84	− 0.05	**22.5**
Left optic radiation	**1.84**	0.43	0.86	1.37	− 0.78	− 1.39	**11.4**
Right optic radiation	**2.47**	**2.53**	− 0.39	0.61	− 0.02	0.53	**16.6**
Left corona radiata	**4.80**	**4.26**	**3.36**	2.01	0.78	− 1.04	**27.0**
Right corona radiata	**4.05**	**4.42**	**2.59**	1.67	− 0.11	− 1.47	**26.6**

**Table 4:** Summary of age-corrected partial correlations (T-statistics) between MWF and R1 (1/T_1_) over different brain white matter regions and pathways. Bold type denotes significant (p < 0.05, uncorrected) correlations.

R1						
Region/tract	0–6 months (N = 25; critical T = 2.06)	6–12 months (N = 26; critical T = 2.06)	12–24 months (N = 35; critical T = 2.03)	24–36 months (N = 23; critical T = 2.06)	36–48 months (N = 11; critical T = 2.20)	48–60 months (N = 35; critical T = 2.03)
**Body (corpus callosum)**	1.11	− 0.92	**2.62**	0.38	− 0.15	0.13
**Genu (corpus callosum)**	0.80	− 0.85	1.89	0.96	− 1.24	1.57
**Splenium (corpus callosum)**	1.36	− 0.96	0.56	− 0.70	− 0.90	0.40
**Left cerebellar white matter**	1.02	− 0.42	− 0.79	0.48	1.35	− 0.09
**Right cerebellar white matter**	0.93	− 0.66	0.86	1.46	0.69	− 0.26
**Left frontal white matter**	1.88	− 0.69	**2.63**	− 0.37	− 0.04	1.55
**Right frontal white matter**	1.12	− 0.75	**4.26**	− 0.17	**− 2.41**	0.71
**Left parietal white matter**	1.64	− 1.41	1.12	− 0.61	0.69	0.05
**Right parietal white matter**	1.57	− 0.87	**2.43**	− 1.43	0.12	0.17
**Left occipital white matter**	1.33	− 1.23	0.09	1.08	**2.66**	1.00
**Right occipital white matter**	1.62	− 1.32	− 0.96	0.01	1.52	0.51
**Left temporal white matter**	1.53	**− 2.10**	1.05	0.07	2.66	0.08
**Right temporal white matter**	0.74	**− 2.06**	**2.69**	− 1.28	1.29	− 0.89
**Left cingulum**	1.26	− 1.30	**2.32**	0.68	− 0.88	0.56
**Right cingulum**	0.59	− 0.15	**2.78**	0.73	− 1.11	1.25
**Left internal capsule**	1.04	− 0.59	**2.65**	0.72	2.01	− 0.17
**Right internal capsule**	0.78	− 0.79	**4.42**	− 0.08	**2.37**	− 0.37
**Left optic radiation**	0.94	**− 2.08**	0.09	0.40	**2.55**	0.82
**Right optic radiation**	1.12	− 1.26	0.96	− 0.15	0.97	− 0.29
**Left corona radiata**	1.89	− 1.02	**3.35**	− 0.13	1.32	− 0.13
**Right corona radiata**	1.41	− 1.08	**4.37**	0.47	0.74	1.10

**Table 5:** Summary of age-corrected partial correlations (T-statistics) between MWF and R2 (1/T_2_) over different brain white matter regions and pathways. Bold type denotes significant (p < 0.05, uncorrected) correlations.

R2						
Region/tract	0–6 months (N = 25; critical T = 2.06)	6–12 months (N = 26; critical T = 2.06)	12–24 months (N = 35; critical T = 2.03)	24–36 months (N = 24; critical T = 2.06)	36–48 months (N = 11; critical T = 2.20)	48–60 months (N = 21; critical T = 2.08)
Body (corpus callosum)	− 0.34	0.12	− 1.54	− 0.67	0.27	1.20
Genu (corpus callosum)	− 0.89	− 0.06	− 1.53	− 0.38	− 0.38	**2.24**
Splenium (corpus callosum)	1.11	0.39	− 0.34	− 0.67	0.17	0.96
Left cerebellar white matter	− 0.60	0.13	0.11	− 0.08	− 0.38	0.55
Right cerebellar white matter	0.47	0.34	0.33	− 0.21	− 0.34	0.71
Left frontal white matter	0.73	− 0.31	− 1.59	− 0.13	− 0.69	1.78
Right frontal white matter	0.70	0.56	− 0.13	− 1.78	− 0.13	1.90
Left parietal white matter	0.13	1.34	− 0.59	− 0.91	− 0.05	1.03
Right parietal white matter	0.64	1.32	1.18	− 0.86	− 1.18	0.33
Left occipital white matter	− 0.13	1.14	0.32	− 0.26	0.20	1.07
Right occipital white matter	0.10	0.86	1.04	0.09	0.12	0.23
Left temporal white matter	− 0.58	1.10	− 0.59	0.67	− 1.37	0.14
Right temporal white matter	− 0.78	2.04	− 0.02	− 1.40	− 0.10	− 0.01
Left cingulum	− 0.24	1.06	− 1.43	− 0.04	0.14	1.21
Right cingulum	0.11	0.51	− 1.89	− 0.14	− 1.21	1.78
Left internal capsule	− 0.52	− 0.70	− 0.97	− 0.30	0.43	0.37
Right internal capsule	0.41	− 0.63	0.43	− 1.39	1.67	0.11
Left optic radiation	0.71	1.49	0.31	− 1.01	0.66	0.80
Right optic radiation	0.40	0.69	0.99	0.39	− 0.04	− 0.60
Left corona radiata	0.19	− 0.79	− 0.11	− 1.00	− 0.12	0.47
Right corona radiata	0.68	− 0.82	1.08	− 0.64	0.30	1.24

**Table 6:** Calculated logarithmic fits to each brain region for each gender. An F-test was used to determine if the data justified modeling the data independently by gender. Values in bold type denote regions were the male and female data were significantly different (p < 0.05 uncorrected).

Region/tract	Male equation	Female equation	F stat
Left frontal WM	0.06 ∗ ln(Age) − 0.2926	0.057 ∗ ln(Age) − 0.2739	**19**
Right frontal WM	0.0603 ∗ ln(Age) − 0.295	0.0572 ∗ ln(Age) − 0.2759	0.487
Left parietal WM	0.0576 ∗ ln(Age) − 0.2727	0.0549 ∗ ln(Age) − 0.2555	0.441
Right parietal WM	0.0577 ∗ ln(Age) − 0.2716	0.0552 ∗ ln(Age) − 0.2571	0.397
Left occipital WM	0.05158 ∗ ln(Age) − 0.2318	0.0501 ∗ ln(Age) − 0.2228	0.119
Right occipital WM	0.0543 ∗ ln(Age) − 0.2495	0.0526 ∗ ln(Age) − 0.2388	0.124
Left temporal WM	0.0617 ∗ ln(Age) − 0.3001	0.0564 ∗ ln(Age) − 0.2689	**16.92**
Right temporal WM	0.0592 ∗ ln(Age) − 0.2881	0.0564 ∗ ln(Age) − 0.2702	0.38
Left cerebellum WM	0.0411 ∗ ln(Age) − 0.1334	0.0391 ∗ ln(Age) − 0.1187	0.293
Right cerebellum WM	0.0418 ∗ ln(Age) − 0.1383	0.0394 ∗ ln(Age) − 0.121	0.294
Left cingulum	0.059 ∗ ln(Age) − 0.2888	0.0548 ∗ ln(Age) − 0.2634	1.02
Right cingulum	0.0583 ∗ ln(Age) − 0.2838	0.0544 ∗ ln(Age) − 0.2609	1.09
Left corona radiata	0.0701 ∗ ln(Age) − 0.3289	0.0685 ∗ ln(Age) − 0.319	0.001
Right corona radiata	0.0712 ∗ ln(Age) − 0.3345	0.0689 ∗ ln(Age) − 0.3211	0.228
Left internal capsule	0.0535 ∗ ln(Age) − 0.2281	0.0538 ∗ ln(Age) − 0.2313	0.031
Right internal capsule	0.0538 ∗ ln(Age) − 0.2312	0.054 ∗ ln(Age) − 0.2326	0.001
Left optic radiation	0.0603 ∗ ln(Age) − 0.2756	0.059 ∗ ln(Age) − 0.268	0.098
Right optic radiation	0.0603 ∗ ln(Age) − 0.2777	0.0577 ∗ ln(Age) − 0.262	**12.25**
Body of corpus callosum	0.0639 ∗ ln(Age) − 0.3039	0.0613 ∗ ln(Age) − 0.2877	0.402
Genu of the corpus callosum	0.0699 ∗ ln(Age) − 0.342	0.0665 ∗ ln(Age) − 0.3212	**33.16**
Splenium of the corpus callosum	0.0587 ∗ ln(Age) − 0.2716	0.0587 ∗ ln(Age) − 0.2716	0

**Table 7:** Calculated logarithmic fits to each brain region for each hemisphere. An F-test was used to determine if the data justified modeling the data independently. Values in bold type denote regions were the right and left hemisphere data were significantly different (p < 0.05 uncorrected).

Region/tract	Right hemisphere equation	Left hemisphere equation	F stat
Frontal WM	0.059 ∗ ln(Age) − 0.2871	0.0588 ∗ ln(Age) − 0.2849	0.171
Parietal WM	0.0567 ∗ ln(Age) − 0.2658	0.0565 ∗ ln(Age) − 0.2654	0.0547
Occipital WM	0.0509 ∗ ln(Age) − 0.2281	0.0536 ∗ ln(Age) − 0.2451	1.007
Temporal WM	0.058 ∗ ln(Age) − 0.2806	0.0596 ∗ ln(Age) − 0.2876	0.605
Cerebellum WM	0.0407 ∗ ln(Age) − 0.1308	0.0402 ∗ ln(Age) − 0.1269	0.037
Cingulum	0.0568 ∗ ln(Age) − 0.2746	0.0573 ∗ ln(Age) − 0.2784	0.038
Corona radiata	0.0703 ∗ ln(Age) − 0.3291	0.0695 ∗ ln(Age) − 0.3248	0.073
Internal capsule	0.0539 ∗ ln(Age) − 0.2318	0.0536 ∗ ln(Age) − 0.2296	0.019
Optic radiation	0.0593 ∗ ln(Age) − 0.2713	0.0598 ∗ ln(Age) − 0.2713	0.136

## Appendix A Age-specific mcDESPOT imaging protocols

### 3 to 9 months of age

14 cm × 14 cm × 13 cm Sagittal Field of View (FOV) with an 80 × 80 × 72 acquisition matrix.

SPGR: echo time (TE)/repetition time (TR) = 5.8 ms/12 ms; flip angles = {2, 3, 4, 5, 7, 9, 11, 14} degrees; receiver bandwidth (BW) = 350 Hz/voxel; 6/8 partial k-space in the phase and slice‐encode directions.

IR-SPGR: matched to SPGR with inversion times of 600 ms and 950 ms; half the resolution in the slice direction. 6/8 partial k-space acquisition in the phase-encode direction.

bSSFP: TE/TR = 5 ms/10 ms; flip angles = {9, 14, 20, 27, 34, 41, 56, 70} degrees; receiver bandwidth (BW) = 350 Hz/voxel; 6/8 partial k-space in the phase and slice–encode directions. Two sets of bSSFP data are acquired with phase-cycling increments of 0 and 180°.

Total acquisition time = 18:22.

Unprotected dB at front of scanner bore: 54 dB.

### 9 to 16 months of age

17 cm × 17 cm × 14.4 cm Sagittal Field of View (FOV) with an 96 × 96 × 80 acquisition matrix.

SPGR: TE/TR = 5.9 ms/12 ms; flip angles = {2, 3, 4, 5, 7, 9, 11, 14} degrees; BW = 350 Hz/voxel; 5/8 partial k-space in the phase and slice–encode directions.

IR-SPGR: matched to SPGR with inversion times of 600 ms and 900 ms; half the resolution in the slice direction. 6/8 partial k-space acquisition in the phase-encode direction.

bSSFP: TE/TR = 5.1 ms/10.2 ms; flip angles = {9, 14, 20, 27, 34, 41, 56, 70} degrees; BW = 350 Hz/voxel; 5/8 partial k-space in the phase and slice‐encode directions. Two sets of bSSFP data are acquired with phase-cycling increments of 0 and 180°.

Total acquisition time = 18:42.

Unprotected dB at front of scanner bore: 62 dB.

### 16 to 28 months of age

18 cm × 18 cm × 15 cm Sagittal Field of View (FOV) with an 100 × 100 × 88 acquisition matrix.

SPGR: TE/TR = 5.4 ms/12 ms; flip angles = {2, 3, 4, 5, 7, 9, 11, 14} degrees; BW = 350 Hz/voxel; 5/8 partial k-space in the phase and slice‐encode directions.

IR-SPGR: matched to SPGR with inversion times of 500 ms and 850 ms; half the resolution in the slice direction. 6/8 partial k-space acquisition in the phase-encode direction.

bSSFP: TE/TR = 5 ms/10 ms; flip angles = {9, 14, 20, 27, 34, 41, 56, 70} degrees; BW = 350 Hz/voxel; 5/8 partial k-space in the phase and slice‐encode directions. Two sets of bSSFP data are acquired with phase-cycling increments of 0 and 180°.

Total acquisition time = 21:38.

Unprotected dB at front of scanner bore: 69 dB.

### 28 to 48 months of age

20 cm × 20 cm × 15 cm Sagittal Field of View (FOV) with an 112 × 112 × 88 acquisition matrix.

SPGR: TE/TR = 5.2 ms/11 ms; flip angles = {2, 3, 4, 5, 7, 9, 12, 16} degrees; BW = 350 Hz/voxel; 5/8 partial k-space in the phase and slice‐encode directions.

IR-SPGR: matched to SPGR with inversion times of 500 ms and 800 ms; half the resolution in the slice direction. 6/8 partial k-space acquisition in the phase-encode direction.

bSSFP: TE/TR = 4.4 ms/9.8 ms; flip angles = {9, 14, 20, 27, 34, 41, 56, 70} degrees; BW = 350 Hz voxel; 5/8 partial k-space in the phase and slice‐encode directions. Two sets of bSSFP data are acquired with phase-cycling increments of 0 and 180°.

Total acquisition time = 24:20.

Unprotected dB at front of scanner bore: 74 dB.

### 48 + months of age

20 cm × 20 cm × 16.5 cm Sagittal Field of View (FOV) with an 112 × 112 × 92 acquisition matrix.

SPGR: TE/TR = 4.8 ms/10 ms; flip angles = {3, 4, 5, 6, 7, 9, 13, 18} degrees; BW = 350 Hz/voxel; 6/8 partial k-space in the phase and slice‐encode directions.

IR-SPGR: matched to SPGR with inversion times of 450 ms and 750 ms; half the resolution in the slice direction. 6/8 partial k-space acquisition in the phase-encode direction.

bSSFP: TE/TR = 5 ms/10 ms; flip angles = {9, 14, 20, 27, 34, 41, 56, 70} degrees; receiver bandwidth (BW) = 350 Hz/voxel; 6/8 partial k-space in the phase and slice‐encode directions. Two sets of bSSFP data are acquired with phase-cycling increments of 0 and 180°.

Total acquisition time = 22:45.

Unprotected dB at front of scanner bore: 82 dB.
