# Supplementary material for: Investigating white matter development in infancy and early childhood using myelin water faction and relaxation time mapping
Source: Neuroimage. 2012 Nov 15;63(3):1038–53. doi: 10.1016/j.neuroimage.2012.07.037 (PMC3711836; doi:10.1016/j.neuroimage.2012.07.037)
Supplement: Supplementary file 1 — Supplementary materials. [file mmc1.pdf]

From 2 to 3: Introducing a Third 'Free' Water Component into mcDESPOT

Sean CL Deoni
School of Engineering, Brown University, Providence RI.

INTRODUCTION: Rapid and reliable assessment of myelin content through multi-component analysis of relaxation data (MCR) may provide salient information in de-myelinating disease (such as multiple sclerosis), as well as neurological disorders arising from altered brain connectivity. T_2-based MCR analysis [1] consistently reveals at least 2 water compartments in brain parenchyma, attributed to water between the myelin bilayers; and intra/extra-axonal (IE) water [2]. A third pool, bulk free water (e.g., cerebral spinal fluid, CSF) may also be present [2]. Multi-component Driven Equilibrium Single Pulse Observation of T_1 & T_2 (mcDESPOT) [3] is a rapid, full-brain alternative MRC method that, unfortunately, strictly assumes a two water pool model. While informative, this model may fail outside of 'pure' tissue, i.e., within voxels containing tissue *and* CSF, or within MS 'black holes'; with mcDESPOT likely under-estimating the myelin water fraction (MWF). Inclusion of a third, non-exchanging component to the mcDESPOT model may correct for these effects. (Fig. 1). Here we outline the mathematical framework and investigate results from a healthy adult and an elderly patient with Alzheimer's Disease (AD).

METHODS: The three-component SPGR and bSSFP magnetizations are given by: $M_{SPGR}^{SS} = M_{SPGR}\left(I - e^{A_{SPGR}\times TR}\right)\sin\alpha \times \left(I - e^{A_{SPGR}\times TR}\cos\alpha\right)^{-1}$ and $M_{bSSFP}^{SS} = \left(e^{A_{bSSFP}\times TR} - I\right)A_{bSSFP}^{-1}C \times \left[I - e^{A_{bSSFP}\times TR}R(\alpha)\right]^{-1}$, where *I* is the 3x3 or 9x9 identity matrix, M_{SPGR}, M_{bSSFP}, A_{SPGR}, A_{bSSFP}, *C* and $R(\alpha)$ are 3x3 (SPGR) or 9x9 (bSSFP) matrices containing the relative volume fraction, relaxation, off-resonance, exchange rate, and excitation flip angle (α) terms.

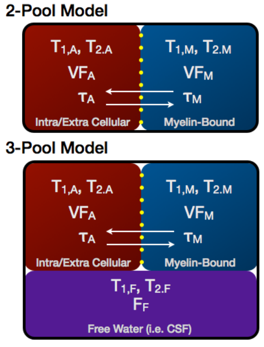

Figure 1: Illustrations of the 2 and 3-pool models

$$A_{SPGR} = \begin{bmatrix} -\frac{1}{T_{1,M}} - k_{M\to IE} & k_{IE\to M} & 0 \\ k_{M\to IE} & -\frac{1}{T_{1,IE}} - k_{IE\to M} & 0 \\ 0 & 0 & -\frac{1}{T_{1,F}} \end{bmatrix}$$

$$C = \rho\begin{bmatrix} 0 & 0 & 0 & 0 & 0 & 0 & \frac{M_F}{T_{1,M}} & \frac{IE_F}{T_{1,IE}} & \frac{F_F}{T_{1,F}} \end{bmatrix}^T$$

$$A_{bSSFP} = \begin{bmatrix} -\frac{1}{T_{2,M}} - k_{M\to IE} & k_{IE\to M} & 0 & \Delta\omega & 0 & 0 & 0 & 0 & 0 \\ k_{M\to IE} & -\frac{1}{T_{2,IE}} - k_{IE\to M} & 0 & 0 & \Delta\omega & 0 & 0 & 0 & 0 \\ 0 & 0 & -\frac{1}{T_{2,F}} & 0 & 0 & \Delta\omega & 0 & 0 & 0 \\ -\Delta\omega & 0 & 0 & -\frac{1}{T_{2,M}} - k_{M\to IE} & k_{IE\to M} & 0 & 0 & 0 & 0 \\ & -\Delta\omega & 0 & k_{M\to IE} & -\frac{1}{T_{2,IE}} - k_{IE\to M} & 0 & 0 & 0 & 0 \\ & & -\Delta\omega & 0 & 0 & -\frac{1}{T_{2,F}} & 0 & 0 & 0 \\ 0 & 0 & 0 & 0 & 0 & 0 & -\frac{1}{T_{1,M}} - k_{M\to IE} & k_{IE\to M} & 0 \\ 0 & 0 & 0 & 0 & 0 & 0 & k_{M\to IE} & -\frac{1}{T_{1,IE}} - k_{IE\to M} & 0 \\ 0 & 0 & 0 & 0 & 0 & 0 & 0 & 0 & -\frac{1}{T_{1,F}} \end{bmatrix}$$

Fitting of this model is performed via the stochastic region contraction approach outlined previously [4].
In Vivo sagittal adult data were acquired as: $22cm^2$x17cm FOV; 128x128x98 matrix. *SPGR*: TE/TR=2.4ms/5.4ms; α=(3, 4, 5, 6, 7, 9, 13 and 18)°; BW = 380 Hz/voxel *bSSFP:* TE/TR=2.2ms/4.4ms; α=(10, 13, 17, 20, 23, 30, 43, and 60)°; BW=560 Hz/voxel. A reduced resolution IR-SPGR image was also acquired (TI=450ms), and the bSSFP data were acquired with 0 and 180° RF phase-cycling patterns [4]. Following mcDESPOT processing [4], 2 and 3-pool models were fit to the data and the resultant MWF maps qualitatively compared.

RESULTS & CONCLUSIONS: Result 2-pool MWF and 3-pool MWF and bulk water fraction maps are shown in Fig. 2. Figure 3 shows a subtraction of the 3-pool model from the 2-pool model results. Marked increases in MWF (in some areas more 50%) are observed at the boundaries the ventricles and periphery of cortical grey matter in the 3-pool results. Within the 'deeper' grey and white matter, less than 3% difference is seen between the 2 and 3-pool models. In the AD patient results, the free water pool is also observed within the altered white matter. These results suggest the original 2-pool mcDESPOT implementation may be insufficient in specific brain regions or pathologically altered tissue, yielding a biased and under-estimated MWF value. Qualitatively, the 3-pool approach appears to correct for these issues, and may be a more suitable choice for investigations of disease.

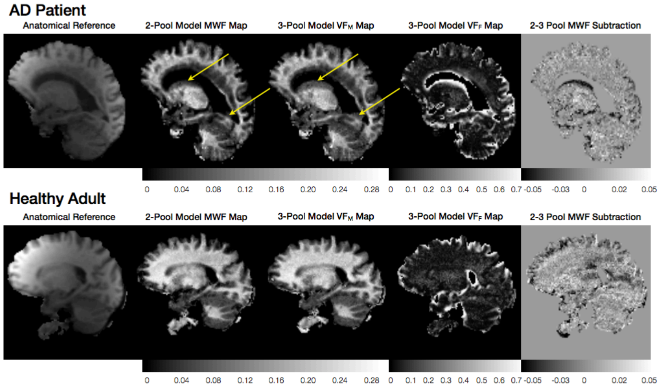

Figure 2: *In vivo* results from a healthy adult and an eldery patient with Alzheimer's Disease (AD). Significant CSF volume fraction is apparent surrounding the ventricles and the periphery of the brain.

REFERENCES: [1] Kroeker RM, Henkelman RM. J. Magn Reson. 1986; 69: 218-235. [2] Whittal KP, et al. Magn. Reson. Med. 1997; 37: 34-43. [3] Deoni SCL, et al. Magn. Reson. Med. 2008; 60: 1372-1387. [4] Deoni SCL. Magn. Reson. Med. 2011; 65: 1021-1035.
